# Pope Francis, climate message, and meat tax: evidence from survey experiment in Italy

**DOI:** 10.1038/s44168-023-00040-x

**Published:** 2023-05-04

**Authors:** Nela Mrchkovska, Nives Dolšak, Aseem Prakash

**Affiliations:** 1 Department of Political Science, Center for Environmental Politics, University of Washington, Seattle, USA; 2 Sustainability Science, School of Marine and Environmental Affairs, University of Washington, Seattle, USA; 3 Department of Political Science, Walker Family Professor for the College of Arts and Sciences, UW Center for Environmental Politics, University of Washington, Seattle, USA

**Keywords:** Attribution, Politics

## Abstract

The livestock sector accounts for 14.5% of global greenhouse emissions. Using an online survey experiment (*n* = 1200) in Italy, we examine respondents’ willingness to support a public petition for a meat tax sponsored by a nongovernmental organization (NGO) after priming them with information on the environmental impact of meat and an embedded moral message. Aiming to test whether institutional authority enhances the appeal of the moral message, we include Pope Francis (a religious authority) and a Professor of Philosophy (a secular authority) as the treatment frames along with a no-messenger (control) frame. Overall, support for meat tax is not significant in any of the treatment frames. However, highly religious individuals (those that practice and intensely believe in religion) across denominations and frames are more supportive of the meat tax. Moreover, we also find that there is a slight backlash among highly religious individuals when they receive the message with the Pope as messenger.

## Introduction

Can moral appeals from authority figures influence individual-level climate preferences, including their willingness to incur private cost to support climate advocacy? Much of the media attention in climate policy focused on fossil fuels. Consequently, policy debates center around how to decarbonize the electricity sector (by using renewables instead of coal and gas) and the transportation sector (by replacing the internal combustion engine with electric vehicles). Less attention is devoted to the agricultural industry, which accounts for approximately 26% of greenhouse gas (GHG) emissions^1,2^. Within the agricultural sector, livestock production (including dairy and meat industries) contributes to 14.5% of GHG emissions^3^. Given dietary trends across the world, the meat industry’s contribution to global GHG emissions will substantially increase by 2050. This is why scholars note the need to change dietary habits, from meat-based proteins to plant-based ones^4–10^.

Meat consumption has an important cultural dimension, which makes changing dietary habits difficult^11^. Yet, if meat consumption is sensitive to meat prices, an appropriate level of a meat tax could incentivize individuals to consume less meat. Indeed, meat tax as a climate mitigation policy has gained some attention in the German and Danish policy debates^12,13^. Scholars have also suggested that this tax should reflect the carbon footprints of different types of meats^14^. After all, in terms of per gram of protein, beef’s climate footprint is six times that of pork’s, and eight times that of poultry’s^2^. Thus, a GHG-weighted meat tax would address the varying climate impact of different types of meats.

Countries use a large number of policy instruments to encourage actors to reduce GHG emissions. These include command and control (such as the U.S. Clean Power Plan), renewable portfolio standards, cap-and-trade, carbon taxes, and information-based policies. While public support for hypothetical climate policy (where cost individual will incur are not clearly stated) seems robust, there is sometimes a pushback when governments seek to impose climate-related taxes; consider the “yellow jacket” (*gilets jaunes*) protest in France^15^ or the failure of carbon tax referendums in Washington state^16^.

Suppose an authority figure takes a public position about the moral dimension of climate change. Might this influence public support for a climate tax^17^? Pope Francis has been among the most outspoken advocates of climate action, frequently addressing the threats of climate change to the environment and humanity and the moral imperative to address them. (For example, see Pope Francis’ famous letter called *Laudato Si’*: https://www.vatican.va/content/francesco/en/messages/pont-messages/2021/documents/20210901-messaggio-protezionedelcreato.html.) Scholars have investigated whether his public statements and, most notably his encyclical letter *Laudato Si’*, have shaped public attitudes and constructed norms towards climate change^18–22^. Yet, it is not clear whether the public is responding to the moral message per se or to the moral message enhanced with Pope Francis’s institutional authority (the *Francis Effect*).

To investigate these issues, we examine the support for a meat tax in Italy. Arguably, because meats are culturally important in Italian cuisine, Italians might therefore be less willing to support a meat tax. (Italy is in the top decile among countries regarding per capita meat consumption. Along with cultural reasons, this probably also reflects the fact that meat consumption correlates highly with per capita income^23^.) Yet, Italy is also a deeply Catholic country, where Pope’s moral message on climate change is likely to resonate. Moreover, unlike the U.S., where Catholics are divided over Pope Francis’ climate advocacy due to political polarization, Italy has witnessed such controversy to a lesser degree, reducing the need to place political convictions at the center stage in assessing Pope’s moral appeal on climate issues^19^. Thus, the Italian case presents an interesting conundrum because it reflects strong reasons why a meat tax policy endorsed by the Pope might get strong support, as well as why it might face strong resistance due to meat’s cultural importance.

To test support for the meat tax in response to a moral message, with and without an institutional authority, and specifically to test the *Francis Effect*, we conducted a survey experiment in Italy. (To clarify, we are *not* testing the appeal of a moral message versus a scientific message on climate issues. Instead, we focus on whether the appeal of moral messages gets enhanced when an institutional authority is invoked.) We asked about individuals’ willingness to donate €20 to a nongovernmental organization (NGO) that is advocating for a meat tax. Climate action is sometimes impeded by individual-level belief in their “causal inefficacy”^24^, that is, individual action cannot influence public policy. NGOs could be an antidote to causal efficacy because they provide a collective voice to a large number of people. NGOs, as preference aggregators, could help individuals recognize that there are others who share their political beliefs and are willing to act on them. Thus, we suggest asking for support by signaling collective voice, rather than an individual support of the tax, which is a more realistic channel of action. Moreover, instead of asking individuals for their endorsement of NGO advocacy, we asked for the willingness to donate a non-trivial but realistic sum of €20. (Future research should test actual donations as opposed to the willingness to donate.) By doing this, we present respondents with a realistic scenario since NGO advocacy is often supported through donations. Moreover, this ask hopefully elicits more thuthful answers because individuals are asked to hypothetically incur private costs to support collective action for a public good. Hence, we investigate individual willingness to donate to an NGO that is advocating for a meat tax.

To test the effects of institutional authority (and to isolate the *Francis Effect* more explicitly), we contrast the appeal of a moral message on its own (in the reference frame the message is not attributed to an authority figure) with a moral message attributed to Pope Francis (religious authority figure), and to Philosophy Professor Giannelli (secular authority figure). Much to our surprise, in relation to the reference category, we find that messenger attributes (religious or secular) do not influence support for a meat tax. Furthermore, the support meat tax does not change *even among* Catholics when the meat tax message is attributed to the Pope. Yet, using a well-established religiosity index (which has three components: the importance of religion in one’s life, frequency of attendance in religious services, and the frequency of prayer), we find that intensely religious individuals show higher support for meat tax relative to those that score lower on the religious intensity scale, but moreover, highly religious individuals tend to be less supportive when primed with the Pope frame relative to the control frame, suggesting a slight backlash effect in line with previous findings^19^. Overall, our results suggest that while religious affiliation does not have an effect on the support for a meat tax, practicing and valuing religion matters across all three frames. Religious individuals respond to the moral message with or without institutional authority and those that rank highly on the religious intensity scale resist the Pope as messenger more than those that rank lower.

Finally, we also test the willingness to support the tax across the whole sample and find that individuals that perceive NGOs to be effective, that believe NGOs work per their stated objectives, and that governments effectively use tax revenue are more likely to support the tax. The same holds for individuals that rank climate change as one of the top two issues facing Italy (among others such as unemployment, inflation, crime, COVID-19, immigration, crime, corruption, terrorism, and social security). Lastly, in terms of individual attributes, we find that frequency of eating meat *does not* influence support for the meat tax, but individuals concerned about meat’s health effects are more likely to support the tax. Those concerned that the tax would harm exports are less likely to support the meat tax as are low-income respondents (incomes below 35k euros/year) and female respondents.

### Literature survey and study expectations

Climate change is a complex policy problem involving both global collective action issues^25^ and domestic distributional conflicts^26^. Because climate protection is a global public good, individuals are sometimes unwilling to incur private costs, either directly paying via some sort of a climate tax, or indirectly through behavioral changes, to support its provision. In a recent global poll, while 78% of respondents noted their concern about the climate crisis, half of them didn’t feel the need to change their habits^27^. Climate protection measures are also hampered by the asymmetrical distribution of climate mitigation costs, the core issue in many domestic political conflicts. Sectors and actors that tend to bear a higher burden of mitigation costs have the incentives to mobilize against climate policy, which could be facilitated by organized interest groups^28,29^.

As voters and consumers, individuals can contribute to climate mitigation^30^. Individuals could place climate protection on top of their electoral priorities and support candidates with explicit pro-climate agendas^31^. As consumers, individuals could opt for low carbon footprint products, eating less meat in our case. Scholars have called this “political consumerism”^32^ or “ethical consumerism”^33^. While individuals might be motivated to take pro-climate actions, they may face collective action issues. NGOs, either through political advocacy or through consumption-focused campaigns, could provide the assurance that the issue is supported by a large number of individuals. The credibility of such assurances depends on whether individuals believe that NGOs work per their stated objectives and NGO advocacy could change public policy.

Is individual willingness to support policy measures such as new tax also influenced by climate change’s moral dimension^17,34^ ? When an issue is given moral weight, the importance of the issue is likely to increase^35^. In addition, the value-belief-norm theory^36^ posits that activating moral norms in individuals helps bolster pro-environmental engagement as moral norms influence individual sensitivity to the idea of harming the environment or other individuals and species. Thus, framing climate change as a moral issue may activate moral norms in individuals and has an effect on how individuals perceive and act in pro-environmental manner.

Religious leaders such as Pope Francis and the Dalai Lama, activists such as Greta Thunberg, as well as celebrities such as Leonardo DiCaprio have also emphasized the moral dimension of the climate crisis. The literature has been especially interested in whether Pope Francis’ public statements and his encyclical letter *Laudato Si’* have shaped public attitudes toward climate change, the *Francis Effect*^19,21,22^. Maibach et al.^20^ report that Pope’s 2015 U.S. tour enhanced the concern about climate issues among Americans, and especially Catholic Americans. Schuldt et al.^22^ find that among U.S. respondents, a brief exposure to Pope Francis’ appeal enhanced the perceptions of climate change as a moral issue. Yet, it is not clear whether individuals are responding to the moral message per se or to the religious authority of Pope Francis. Moreover, might institutional authority in general, and not the religious authority of Pope Francis per se that drives the effect^37,38^? Research has shown that policies championed by leaders with high moral character across different message framing are more likely to be supported by individuals^39^. However, it may also be the institutional authority of the individual. Indeed, research finds that when uncertainty is high, the carrier of the message is used as a heuristic in judging the content of a message and policy support^40,41^. Thus, even though Pope Francis, being the head of the Catholic Church, may signal high morality to individuals, it may also be the institutional credibility rather than the moral character of the messenger. For this reason, in this study, we isolate the institutional authority from the message and we focus primarily on investigating whether institutional authority matters in the way moral messages are perceived by individuals, specifically religious and secular moral authority (as this is where the debate on the *Francis Effect* is nested) compared to the effects of the moral message that is not backed by institutional authority. (To make the concept of institutional authority clearer, for example, Greta Thunberg is an activist but without institutional authority. Similarly, Leonardo DiCaprio is a celebrity but without institutional authority. In contrast, Pope Francis represents institutional religious authority, and a university professor represents institutional secular authority.)

Merging these two ways of thinking about pro-environmental attitude appeals, we posit that framing of climate change as a moral issue and having it delivered by an individual with institutional authority will increase support for climate action relative to moral message only. To explore the issue of the “message versus messenger” effect, we propose the following hypotheses:

1. Respondents are more likely to donate to the NGO advocating for a meat tax (support a meat tax henceforth) when primed with a moral message by Pope Francis.
2. Respondents are more likely to support a meat tax when primed with a moral message by a professor of philosophy.

Individual-level characteristics could make respondents more receptive to the moral message from a secular authority or the Pope. For example, might Catholics be more receptive to the moral message from the Pope?^42^ find that framing climate change as a religious issue encourages greater engagement among Christians. Boas^41^ finds that Christians respond more favorably to candidates that have “pastor” in their titles compared to other groups. This suggests that individuals who associate with their groups are more likely to take action which coheres with the preferences of the group’s representative or leader. In our case, Catholics should be more receptive to the moral message from the Pope.

However, scholars have pointed out that religiosity may be a separate theoretical construct with distinct empirical effects, different than religious affiliation^43,44^. We, therefore, differentiate between nominal membership in religious denomination (specifically, Catholicism) and follow scholars who have quantified religiosity in dimensions other than religious nominal affiliation^45,46^. Drawing from these scholars and the broader conception of religion in the literature on the economics of religion^44,47^, we use a well-established “index of religiosity” that reflects three individual-level characteristics: whether individuals view religion to be important in their lives, pray frequently, and attend religious services frequently. We use these three dimensions to capture both internal (importance of religion in one’s life) and cost-based (time and resources spent on attending and praying) dimensions of religious intensity.

The debate on the relationship between religiosity and morality of individuals (i.e., are religious people more moral than non-religious people) is inconclusive. Religion-based morality often centers around harm/care and fairness/reciprocity foundations^48^, both of which are connected to pro-environmental behavior per the value-belief-norm theory^36^. Thus, we expect individuals that rank high on the religiosity scale to be more responsive to Pope’s message given its religious content. Thus, we hypothesize:

3. Respondents who identify as Roman Catholic are more likely to support the meat tax when primed with a moral message by Pope Francis.
4. Respondents with high levels of religiosity are more likely to support the meat tax when primed with a moral message by Pope Francis.

In a well-balanced and randomized experimental design as we adopt in this paper, the different effect of the frames on the support of meat tax should not be confounded by any other variables. However, as a robustness check, we test for alternative mechanisms shaping the relationship between the message framing and pro-environmental attitudes. We include measures on individual perceptions of climate change’s policy salience and institutional effectiveness (of NGOs and their government), perception of others’ actions (or inaction) regarding climate change, and pre-existing individual dietary habits. The theoretical expectation is that any of these mechanisms may influence the support for the meat tax. For example, if individuals do not think that NGOs are effective or if they fear a “tax and spend” government^49^, then they are likely to oppose a meat tax, not because they oppose climate action, but because they do not trust the channels of action. Finally, if individuals believe meat is bad for their health^50^, they will support the meat tax for health reasons, and not for climate concerns. Thus, to rule out confounding factors, we include those measures in our conservative estimation.

### The experiment

In January 2022, we conducted an online survey experiment in Italy using the Qualtrics platform. We engaged a survey company, Cint, to recruit the respondents and administer the survey in Italian language. To ensure linguistic clarity and cultural appropriateness, a native Italian speaker translated the survey from English to Italian, and another native speaker translated it back from Italian to English. This provided us with additional confidence that our survey conveyed the appropriate information to Italian respondents.

Our nationally representative sample consisted of 1200 respondents randomly assigned into three groups: one reference group and two treatment groups. In terms of respondents’ demographics, 60% of the individuals identified as religious and 40% as non-religious. Thus, we have a higher representation of non-religious because nationally, 87% of Italians claim to be religious and 12.7% are not affiliated with any religion. Thus, we reweighted our sample to match the national statistic of religious versus non-religious individuals and use the religion-weighted sample in our analysis in the main analysis, although the results hold for the non-weighted sample as well. (We used the package “survey” in R to add post-stratification weights.)

Among respondents who identify themselves with a religious denomination, 93% are Catholic, which is only slightly higher than the 91% Catholic population in Italy. In terms of age distribution, our respondents are between 18 and 65 years, of which 15% are in the age range between 18 and 30, 23% are between 30 and 40 years old, 33% are between 40 and 50 years old, and 5% are between 50 and 60 years old; the age distribution matches the national one. Our sample is almost equally split among men and women, also approximating the national average. 72% in our sample are employed (full-time, part-time, and self-employed included), 9% were homemakers, and 9% were unemployed but looking for work. Lastly, our sample is nationally representative in terms of region of residence. (Climate change is not a partisan issue in Italy. Hence, we did not seek to match the sample on national-level data on party affiliation or ideology.) Table 1 describes the sample.

**Table 1:** Sample description.

	Group 1 Pope (*N* = 400)	Group 2 Professor (*N* = 400)	Group 3 Control (*N* = 400)	Total Sample (*N* = 1200)
*Gender*				
Male	189	188	194	571 (48%)
Female	208	210	202	620 (52%)
*Education*				
Post-secondary education	96	116	88	300 (25%)
University degree	57	58	57	172 (14%)
High school or lower	247	226	255	728 (61%)
*Employment*				
Employed	290	285	288	863 (72%)
Looking after home	32	40	37	109 (9%)
Looking for job	35	33	35	103 (9%)
Student	20	23	23	66 (5%)
Unemployed/retired	23	19	17	59 (5%)
*Age*				
18–30	55	62	59	176 (15%)
30–40	93	93	87	273 (23%)
40–50	132	139	130	401 (33%)
50–60	92	86	101	279 (23%)
60–65	26	20	23	69 (6%)
*Household Income*				
Above 35k/year	98	112	110	320 (27%)
Between 25–35k/year	127	134	147	408 (34%)
Below 25k/year	174	155	143	472 (39%)
*Marital status*				
Married	222	209	221	652 (54%)
Divorced/separated	17	24	16	87 (7%)
Single	154	154	146	454 (38%)
Widowed	1	2	4	7 (<1%)
*Religious affiliation*				
*Identify with religion*	248	231	238	717 (60%)
Catholic	226	217	226	669 (93%)
Other Christians	13	10	7	31 (4%)
Other religions (Islam, Buddhism, Judaism)	7	3	5	17 (3%)
*Do not identify with religion*	152	169	162	483 (40%)

After the survey was approved by our University’s Human Subjects Division (STUDY00014314), we pre-registered the survey and the analysis plan here https://osf.io/kesjm/. In the analysis, we follow the pre-analysis plan (PAP). Although we test and report each hypothesis listed in the PAP, to shorten and streamline the theoretical discussion, we do not list all of them. After pilot testing the survey, we fully launched the survey in January 2022. In terms of survey design, we randomly assigned respondents into three groups who received information in the form of a hypothetical newspaper article on the meat tax.

**Fig. 1:**
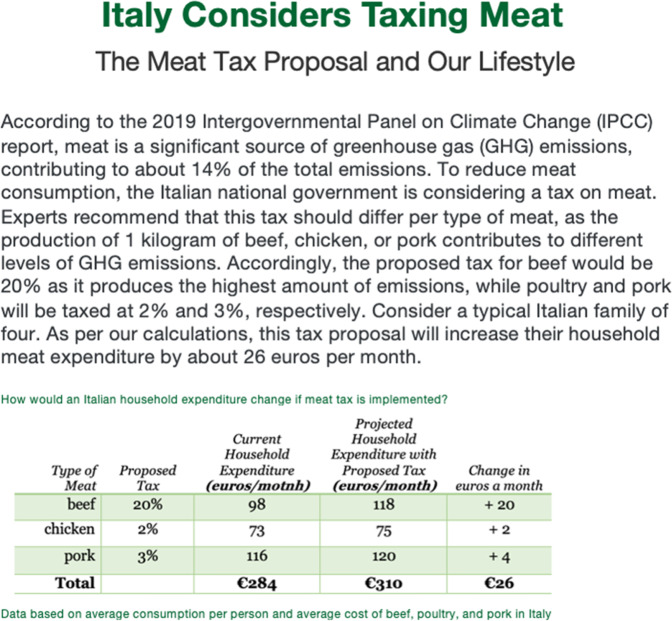
The hypothetical newspaper article that respondents read as part of the survey experiment.

This article had two paragraphs. The first paragraph, which all respondents read, referenced a report by the IPCC and presented factual information about the effect of food production on GHG emissions, and it also provided the rationale for different levels of meat tax (see Fig. 1). Using the estimated optimal GHG-weighted meat tax per type of meat^14^, we combined this information with the average consumption of different meats in Italian households and presented it in an easily readable table that includes information on the pre-tax and the projected (post-tax) household expenditure on meat products for a typical household of four. The reason is that we want individuals to think through the financial implications of a meat tax.

The second paragraph pertained to the moral message. While all respondents read the identical moral message, we randomly varied the messenger across the three groups. Pope Francis was the messenger in the religious authority frame and a professor of philosophy, Professor Giannelli (a common Italian surname) in the secular authority frame. In the reference category, we did not identify any authority figure as the messenger. Table 2 presents the text of the three frames, which have comparable word counts.

**Table 2:** Treatment and control group frames.

**Control frame**
Should Italian families support this tax? We need to be reminded that our land is valuable and show it respect. Everyone ought to “till and keep” the garden of the world by cultivating and ploughing, but also caring, protecting, overseeing, and preserving the land. We need to recognize that people should take whatever they need from the bounty of the Earth for their subsistence. But in the process, we should not forget about our duty to protect the Earth and to ensure its fruitfulness for future generations. While every household will make their own decisions on the recent tax proposal, we ought to be reminded about our responsibilities, think and reconsider our lifestyle and impact on Earth and future generations.
**Religious frame (Pope Francis)**
Should Italian families support this tax? The Encyclical Laudato Si’ by Pope Francis may offer some guidance. In his Encyclical, Pope Francis urges everyone to “till and keep” the garden of the world by cultivating and ploughing, but also caring, protecting, overseeing, and preserving the land. Pope Francis recognizes that people should take whatever they need from the bounty of the Earth for their subsistence. But in the process, he asks us not to forget about our duty to protect the Earth and to ensure its fruitfulness for future generations. While Pope Francis does not directly address the recent tax proposal, his message may guide us as we reconsider our lifestyle and our impact on Earth and future generations.
**Secular frame (Professor Giannelli)**
Should Italian families support this tax? The book How We Live by Professor Giannelli may offer some guidance. In his book, Professor Giannelli urges everyone to “till and keep” the garden of the world by cultivating and ploughing, but also caring, protecting, overseeing, and preserving the land. Professor Giannelli recognizes that people should take whatever they need from the bounty of the Earth for their subsistence. But in the process, he asks us not to forget about our duty to protect the Earth and to ensure its fruitfulness for future generations. While Professor Giannelli does not directly address the recent tax proposal, his message may guide us as we reconsider our lifestyle and our impact on Earth and future generations.

We recognize that online surveys have low-effort and inattentive respondents. Therefore, after reading the hypothetical newspaper article, we asked two attention check questions^51^. Although in the main model we report respondents who answered at least one of the two questions correctly, our results do not change when we include all respondents (Appendix A). Also, we do not find any pattern in attention check failures: of the individuals who failed the test, 20 were in the control group, 23 were in the Pope treatment frame, and 20 were in the Professor frame.

After the attention check questions, we asked respondents how likely they were to donate €20 to a (hypothetical) non-profit organization “For the Future” that seeks to petition the Italian government for a meat tax during the next legislative session. In choosing the donation amount of €20, we consulted several Italians and examined average donations to environmental organizations. (See report on giving in Italy for average donation amount: https://ernop.eu/wp-content/uploads/2017/10/Giving-in-Europe-country-report-Italy.pdf.) We also chose the NGO name in consultation with Italian colleagues to ensure that the NGO sounds credible, and that does not already exist in the Italian society.

We provided a slider (continuous) scale from 1 to 7 and ask respondents how likely they are to make a €20 donation to this NGO, with 1 being not likely and 7 being very likely. Then, we asked a series of questions to help us understand the underlying mechanisms that might have motivated respondents’ willingness to support the meat tax. Following these questions, we asked a series of standard demographic questions about their gender, age, income, education level, employment, and marital status. (As shown in Appendix B, the respondents’ characteristics are balanced across the three groups, indicating that the randomization implemented in administering the survey experiment is successful.)

## Results

To interpret our findings, we test three models: one where the dependent variable is continuous (ranging from 1 to 7), ordinal, and binary where the support levels of 5–7 are categorized as individuals being *likely to support* the meat tax (1), and 1–4 as *not likely* (0). Thus, we estimate three models—ordinary least square (OLS), ordered probit, and logit. In the main text we present the OLS results only, but in the appendix, we show that our substantive results hold when we use logit and ordered probit estimators. To facilitate easier reading, we state our findings in terms of the support for a meat tax instead of the likelihood to donate €20 to an NGO advocating for a meat tax.

As reported in Table 3, among all respondents, the average willingness to donate was 3.4 on a scale from 1 to 7; 38% of the respondents in the control frame, 35% in the Pope frame, and 40% in the professor frame indicated high support (above 4). Table 3 also includes the summary statistics of other variables of interest.

**Table 3:** Summary statistics.

	Control frame		Pope frame		Prof frame		Sample	
	Mean	SD	Mean	SD	Mean	SD	Mean	SD
Donation history	3.4	1.989	3.2	1.977	3.5	2.001	3.364	1.99
Religious intensity index	5.4	5.055	5.5	5.04	5.3	5.163	5.408	5.082
NGO effectiveness	4.2	1.301	4.1	1.292	4.2	1.299	4.194	1.296
NGO objectives	4.2	1.215	4	1.272	4.2	1.215	4.122	1.236
Meat vs health	3.8	1.313	3.8	1.317	3.9	1.253	3.814	1.294
Tax vs export harm	4.7	1.163	4.6	1.231	4.7	1.143	4.663	1.18
Govt effectiveness	3.3	1.322	3.1	1.325	3.3	1.294	3.219	1.31

**Fig. 2:**
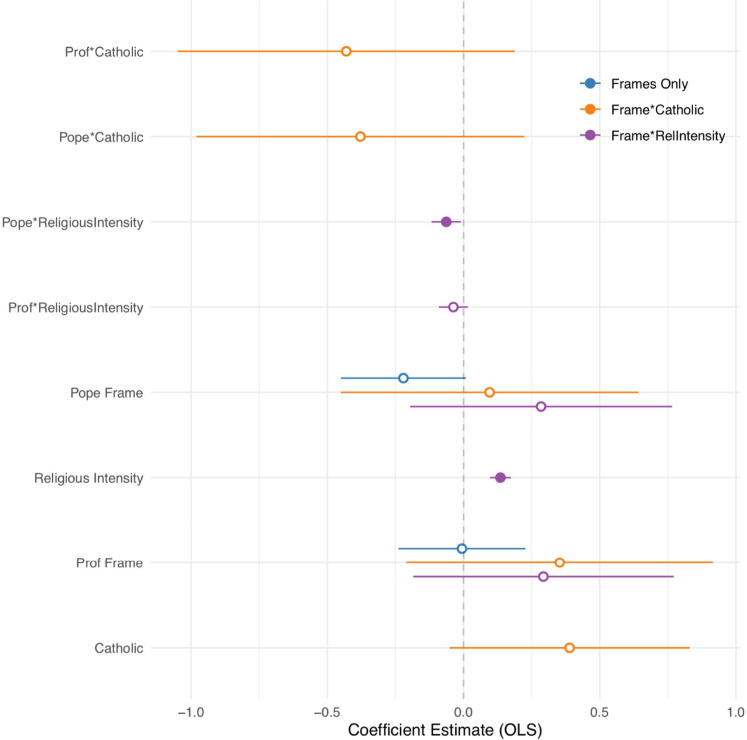
Key results from OLS estimation with and without interaction terms. *Error bars indicate 90% confidence intervals (defined as standard error of the mean).

The estimates across different models and specifications reveal that, *contrary* to our expectations, there is no support for either H1 or H2. That is, in relation to the reference category, respondents are not more likely to support a meat tax when treated with the Pope frame (H1) or secular authority frame (H2). As shown in Fig. 2 (in blue), the statistical difference of the two treatment groups in relation to the control group is not statistically significant, indicating that identifying an authority figure as the moral messenger does not shape respondents’ support for a meat tax. The effect holds across different models (logit, ordered probit results included in the Appendix). Even though our sample is balanced across frames, the Appendix also includes an estimation with additional variables that capture alternative mechanisms. The inclusion of these variables does not alter substantive results about the framing effect. (Coefficients in the Appendix table are estimates after adjustment for the following covariates: trust in NGOs’ effectiveness and objectives, trust in government effectiveness, history of donations to a non-religious organization in the past year, intensity of meat consumption, health concern of meat consumption, concern over export harm from meat tax, ownership of farm, and climate concern.) These findings do not mean that respondents accept or reject the moral framing of the climate crisis—because we are not testing a moral message against a scientific message. Rather, we find that the persuasiveness of the moral messaging does not change even when we invoke a religious authority figure or a secular authority figure.

Is there a *Francis Effect* among Catholics (H3)? To explore this, we interact the Pope frame with the Catholic variable. (We interact with a Catholic dummy only within the religiously affiliated sample, and within the whole sample. The results remain unchanged with both approaches.) We were surprised to find that even among Catholics, the moral messaging from the Pope does not increase the support for a meat tax, notwithstanding Pope Francis’ leadership in the Catholic Church, Italians’ strong affiliation with Catholicism, and the Vatican’s proximity location in Rome. (As Table 3.1 in the Appendix shows, the probit model picks up significance with the Catholic dummy, however, given that this model fares comparatively worse in goodness of fit (according to the AIC measure) and the other two models do not report significance, we leave this finding in the Appendix.) Unfortunately, due to the small sample of non-Catholic religiously affiliated individuals, we are unable to test the effect across other religious traditions.

Individuals might nominally identify with a religion but may not practice or follow the religious principles i.e. “belonging without believing” (McClearly and Barro, 2006). While we do not find that respondents identifying nominally with any religion (or Catholics in particular) supporting a meat tax, might those who practice religion through prayer and attendance do so? We construct a religiosity index that is the sum of scores of three variables (Cronbach’s Alpha = 0.94): the importance of religion in the individual’s life (scale of 0–7), frequency of religious attendance (scale of 0–7), and frequency of prayer (scale of 0–7). The mean value of individuals’ importance of religion across the whole sample is 1.776 (min = 0, max = 4); frequency of religious attendance is 2.51 (min = 0, max = 7); and frequency of prayer is 2.33 (min = 0, max = 6). The overall religiosity index, composed of three questions an scaled across their values, has a mean of 5.4 (min = 0, max = 15) on a scale from 0 to 15. (The religious affiliation and religious index are highly correlated; Pearson’s correlation coefficient is 0.86.)

**Fig. 3:**
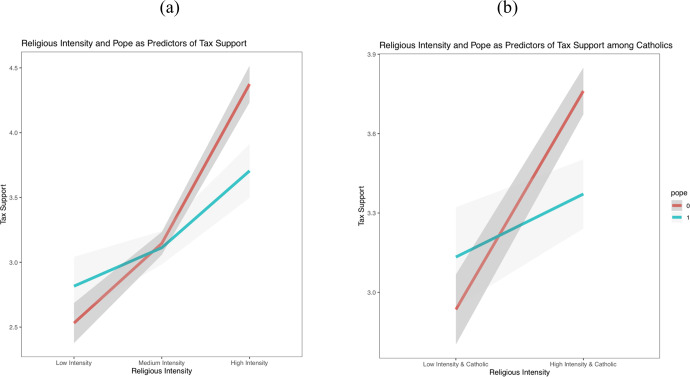
Marginal effects plot. Marginal effects plot of interaction between Pope frame and religious intensity (**a**) and among Catholics (**b**).

We interact the level of religiosity (henceforth, religious intensity) with the frame that the respondent received. While the *Francis Effect* was not significant among respondents who nominally identify themselves as Catholics, is it significant among those who are highly religious, given the universal appeal of the Pope? The Pope frame coefficient remains insignificant, but we find an interaction effect (at 0.1 level and 0.05 significance level in OLS and logit, respectively) between the Pope frame and the religiosity index, indicating a cross-over interaction. The secular frame and interaction term with the secular frame remain insignificant. Figure 3 presents the marginal effects of this interaction and shows that when scoring low on the religious intensity scale, there is no interaction effect with the Pope frame, however, when an individual becomes highly religious i.e., scores above the mean (5.4 on the religious intensity scale), they support the tax more when primed with the control frame relative to the Pope frame. The effect is small, but significant, and it is suggestive of a potential backlash effect to the Pope as the messenger (Fig. 3a). It is likely that this effect is driven mainly by Catholic individuals, given the small sample of other religiously affiliated individuals, thus we also include the results of the interaction between the religious intensity index and highly religious Catholics (by constructing a dummy that equals 1 if the respondent scores above the mean on the religious intensity scale and also identifies as Catholic). We find similar results across Catholic respondents, with an effect difference of 7 percentage points between those primed with control and Pope frame (Fig. 3b).

The results suggest that, in general, as religious intensity of individuals increases, so does their willingness to support the tax. However, among highly religious people and highly religious Catholics, the *Francis Effect* is negative (H4 is not supported). How to explain these findings? The findings that highly religious are more receptive the climate message is suggestive of the theory that religious moralities respond to concerns about harm/care and fairness/reciprocity^48^. Thus, the message itself resonates with individuals that invest personal resources to follow and practice religion^36^. The backlash effect among Catholics is in line with other studies in the literature that find that the Pope (and his message on climate) further polarizes climate change among Catholics, especially in the United States^19^. We find this backlash effect in the Italian context as well. Future work should examine the reasons for this backlash, including religious individuals push back against the involvement of the Pope in, what they consider to be, a political issue^52^, or they question Pope’s competence to address climate change, or meat tax in particular^53,54^.

Considering the sample in its entirety, i.e., not conditional on the framing, we find that individuals that rank climate change as one of the top two issues (among others such as unemployment, inflation, crime, COVID-19, immigration, crime, corruption, terrorism, and social security) facing Italy at the moment show higher support for the tax. However, we do not find higher support among individuals who believe in making an unconditional personal sacrifice to protect the environment, irrespective of what others do.

In terms of trust in institutions, we also find that individuals who trust that the government is using tax revenue efficiently, and who believe in the objectives and effectiveness of NGO show higher support for the meat tax. The level of individual’s meat consumption does not have an effect on the support for the meat tax, but individuals who are concerned about meat’s effect on their health show higher support. We also consider how might the economic status, economic dependency on agriculture, and general economic protectionist tendencies among respondents might affect the support for meat tax. We find that low-income respondents and individuals who believe that the tax will harm domestic producers and exports show lower support for the tax. There is a positive (and slightly significant) result that those who own a farm show higher support for the meat tax relative to those that do not; while this is the opposite of our expectation in terms of cost implication of the tax to farm owners, recent literature finds that rural and urban residents are starting to converge in their support of climate change policies^55,56^. Finally, our results indicate that males support the meat tax about 24 percentage points more than females. This is an important finding because men tend to earn more than women (wage gap) and hence have a higher ability to pay a new tax. However, prior work also suggests that men (especially conservative and white) tend to support climate denialism due to system-justification tendencies^57^^,^^58^. Future work should unpack gender, consider intersectionalities, and examine support for different types of climate action. Table 4 presents the results of this regression.

**Table 4:** OLS regression estimates on full sample.

	*Dependent variable:*
	donation
NGO effectiveness	0.237*** (0.048)
NGO objectives	0.193*** (0.049)
Govt effectiveness	0.336*** (0.044)
History of donations	0.024 (0.106)
Meat consumption	0.040 (0.027)
Meat vs health	0.319*** (0.045)
Tax vs export	−0.265*** (0.044)
Owns a farm	0.330* (0.194)
Climate concern	0.228** (0.113)
Independ. climate action	−0.032 (0.104)
Age (18–29)	0.212 (0.191)
Age (30–39)	−0.378*** (0.141)
Age (40–49)	−0.462*** (0.152)
Age (50–65)	−0.531** (0.242)
Male	0.245** (0.105)
College+	0.074 (0.108)
Steady employment	−0.021 (0.116)
Low income	−0.270** (0.109)
Married	−0.189 (0.126)
Constant	0.418 (0.387)
Observations	1009
R^2^	0.364
Adjusted R^2^	0.352
Residual std. error	1.588 (df = 989)
F Statistic	29.758*** (df = 19; 989)

*p < 0.1; **p < 0.05; ***p < 0.01.

## Discussion

The climate debate has an important moral dimension, specifically the current generation’s responsibility towards future generations. (For example, see the discourse on climate change among public and politicians: https://www.americanprogress.org/article/religious-americans-demand-climate-action/; http://www.vaticannews.cn/en/world/news/2021-05/pope-francis-meets-with-us-envoy-for-climate-john-kerry.print.html.) Yet it is not clear whether individuals respond to the moral message per se, or to the messenger who is delivering it. While holding the moral message constant across the frames, we tested whether support for meat tax changes when a religious authority and secular authority figure are invoked. To our surprise, invoking authority figures does not change support for a meat tax. Surprisingly, we do not find a *Francis Effect* among people belonging to any religion or specifically among Roman Catholics.

Arguably, receptivity to the moral message (especially from Pope Francis) depends not if one is nominally associated with any religion but on whether individuals incorporate religious beliefs in their everyday life or feel connected to god. Indeed, we find that the level of religious intensity has a small but significant effect in support of a meat tax across the frames. It is possible that the religiosity index captures both the socialization role of religion as well as principles about leading a life that is morally sound. This coheres with Schwartz’s value-belief-norm theory, which suggests that religious individuals hold moral norms that make them more likely to engage in pro-environmental behavior^36,59^. While our study does not evaluate why individuals are not responding to the messenger, it finds that individuals with deeply held beliefs about god draw tend to be more supportive of climate action. With this finding, we contribute to the broader literature that investigates religious beliefs and practices as opposed to religious affiliation as predictors of political behavior^60–63^. We also find that there is a slight backlash among highly religious individuals when they receive the message with the Pope as a messenger. While our study does not test for possible mechanisms to explain this backlash, we suggest that highly religious individuals might be pushing back against Pope Francis wading into highly polarized issues which should be outside the purview of the Catholic church or his area of competence. Future research should replicate our research design in the context of the United States, where the climate debate is more polarized, and some Catholic leaders have criticized the Pope’s position on climate issues. (For example, see ref. ^64^ and https://www.ncronline.org/news/vatican/francis-calls-us-catholic-criticism-his-papacy-honor.)

Our study has several implications. Even in societies where meat consumption is culturally important, we find willingness of individuals to incur private costs to address the climate crisis across multiple heterogenous characteristics. Our study reveals that trust in institutions plays an important role in mobilizing support for climate policy. For climate policy to have public support, individuals must trust the government, the main policy supplier. In an era of institutional dysfunctionality and populism^65,66^ where the trust in government has eroded, our findings emphasize that climate discourse cannot be separated from broader concerns about government efficacy. Similarly, while NGOs are important actors in the policy process, across the world, governments are cracking down on NGOs^67^. One factor motivating authoritarian governments is the perception that NGOs sometimes do not enjoy public trust. Scandals in high-profile NGOs such as Oxfam and Save the Children could have eroded public support^68^. Our study thus supports the importance of ensuring that the citizens believe that NGOs work as per their stated objectives and that NGOs can be effective in influencing the policy process.

Our study should also motivate the debate on the usefulness of public opinion surveys in social science research, specifically to what extent expressed attitudes and preferences translate into behaviors. If individual actions are informed by individual attitudes, surveys are helpful. Moreover, the media and policymakers tend to closely follow public opinion surveys, as do business leaders, which enhances the policy salience of the issue and hopefully motivates action. Take the example of reputational rankings, which summarize expert attitudes and play important roles in directing economic flow. Countries make changes in their domestic policies so that they can improve their scores on the “ease of doing business” or “corruption”.

How to differentiate “real attitudes” from cheap talk? There is work on voting issues (validating voting with both pre-election and post-election surveys), as well as validating contingent valuation methods. Some studies find that survey responses match the actual voting outcome^69^. As per our reading, validation depends on how the survey was constructed (administered (face-to-face), telephone, mail, and internet), and how the responses were coded (especially the don’t know/can’t say category in voting surveys)^70^. Broadly, surveys and cheap talk is an important issue^71^, especially with the advent of social media and the enormous pressure to conform to specific norms, along with virtue signaling. This is why we decided that instead of simply asking for the level of support for a meat tax, we should ask respondents to the hypothetical scenario to donate money to support NGOs that are lobbying for a meat tax. The intuition is that this sort of ask will at least encourage respondents to think about private costs they might have to bear in supporting specific policy positions which generate public benefits (Dolšak et al.^72^ made the same argument in the context of assessing public support for a carbon tax).

We recognize that giving actual money and doing a field experiment is an excellent way to observe real-world behavior. However, this approach has drawbacks as well including that the way individuals make the decision about charitable donations varies significantly across households (including the stickiness in charity choices), and asking them to part with a hypothetical amount might change a situation where true preferences might not be revealed. In sum, a multi-methods approach is required to validate specific lessons from public support for a meat tax.

Our study offers several exciting avenues for future research. First, while we do not test the persuasive power of a moral message against a secular message, future work could explore how this issue, including the moral message with and without institutional authority. Further, it could also explore the appeal of secular messages with and without institutional authority (such as the IPCC) and also examine its appeal with a moral frame as a reference. Second, regarding external validity, we chose Italy to observe the *Francis Effect* on support for a meat tax, given its Catholicism, the relatively less controversy about the policy position of the Pope, and the Vatican’s location in Rome. Future research should explore the *Francis Effect* in Catholic countries (such as Ireland or Argentina) and in countries where Catholics are not the majority population (such as Islamic, Buddhist, and Hindu societies). Moreover, regarding the messenger, would we expect a similar finding if say, the Dalai Lama, another prominent religious authority leader who has spoken for climate action, is referenced? Similarly, while meats are an important part of Italian cuisine, might a meat tax find a more supportive audience in societies that are predominantly vegetarian such as India? In addition, meat consumption correlates highly with per capita income^73^; much of the increase in meat consumption is occurring outside Europe and North America. If eating meat is considered as a sign of prosperity, might the pleas for a meat tax be less favorable received in these regions?

Finally, it is important to examine if the experiment component in the survey is sufficiently differentiated across frames (that is, investigate whether the survey might have a “dosage” problem). The treatment frame (the moral message with the messenger) was 57% of the total word count across all frames, while the messenger’s name across the treatment frames was mentioned four times. Future work should investigate whether the results hold if we substantially enhance the salience of the messenger in the survey (for example, the treatment component becomes 75% of the word count, and the messenger is invoked say 8 times instead of 4 in the current instrument). Similarly, while our study focuses on meat consumption, Pope Francis has not been vocal on the issue of meat consumption, and thus, may not be associated with the meat issue. Perhaps another prominent religious leader such as the Dalai Lama who has spoken in favor of vegetarianism might have a different impact on support for a meat tax.

## Methods

The methods were performed in accordance with relevant guidelines and regulations and approved by University of Washington’s Human Subject Division (STUDY##00016191).

In our survey, subjects received the following information regarding written informed consent: “The survey is anonymous. The information you provide will not be stored or used in any way that could reveal your personal identity. There are no known risks posed by participating in this survey. The survey has been reviewed by the University of Washington’s Human Subject Division (STUDY##00016191). Your participation is voluntary, and you may discontinue participating in the survey at any time.”

## Supplementary information

Appendix

## Data Availability

The datasets generated for the current study are posted on the Harvard Dataverse repository, 10.7910/DVN/M9PMKZ and will be made publicly available once the paper is published.

## Code availability

Computer code and algorithm available upon request.
