# Supplementary material for: Pope Francis, climate message, and meat tax: evidence from survey experiment in Italy
Source: NPJ Clim Action. 2023 May 4;2(1):10. doi: 10.1038/s44168-023-00040-x (PMC10158699; doi:10.1038/s44168-023-00040-x)
Supplement: Supplementary file 1 — Appendix [file 44168_2023_40_MOESM1_ESM.docx]

**Appendix A**

Table 1.1: Full Sample Main Model


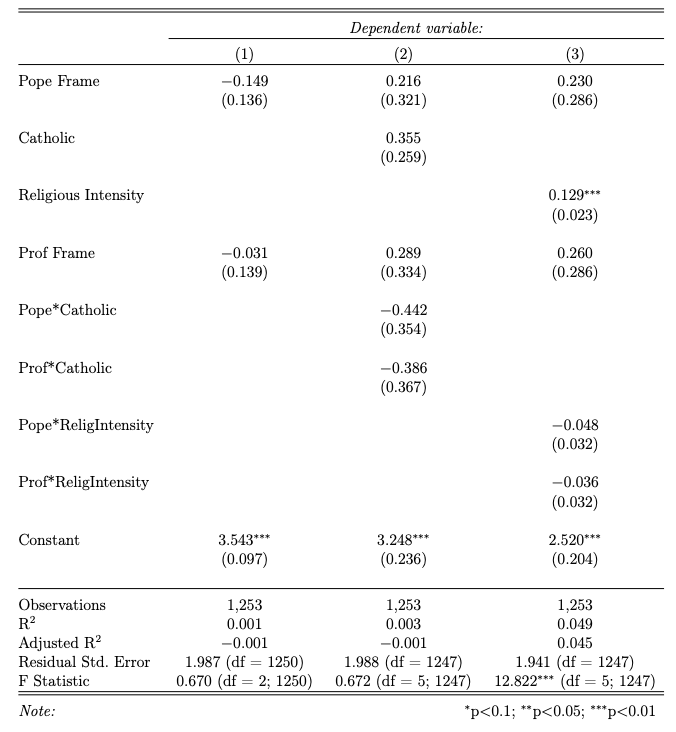


**Appendix B**

Table 2.1: Covariate Balancing

|  | Control | Pope Treatment | Prof Treatment |
| --- | --- | --- | --- |
| **Gender** | | | |
| Male | 48 % | 47 % | 47 % |
| Female | 52 % | 52 % | 52 % |
| **Education** | | | |
| Low education | 64 % | 62 % | 56 % |
| High education | 36 % | 38 % | 44 % |
| **Employment** | | | |
| Employed (Full-time/Self) | 39 % | 36 % | 37 % |
| Other | 61 % | 64 % | 63 % |
| **Age** | | | |
| 18-31 | 16 % | 15 % | 17 % |
| 32-51 | 56 % | 57 % | 59 % |
| over 51 | 27 % | 28 % | 23 % |
| **Household Income** | | | |
| High | 27 % | 25 % | 28 % |
| Medium | 37 % | 32 % | 34 % |
| Low | 36 % | 43 % | 38 % |
| **Marital Status** | | | |
| Married | 36 % | 39 % | 38 % |
| Single (and other) | 64 % | 61 % | 62 % |
| **Religious Affiliation** | | | |
| Identify with religion | 60 % | 62 % | 58 % |
| Do not identify with religion | 40 % | 38 % | 42 % |
| **Owning a Farm** | | | |
| Yes | 6 % | 9 % | 8 % |
| No | 94 % | 91 % | 92 % |
| **History of Donations** | | | |
| Yes | 54 % | 49 % | 50 % |
| No | 46 % | 51 % | 50 % |
| **Independent/Conditional Support** | | | |
| Independent | 61 % | 59 % | 61 % |
| Conditional | 39 % | 41 % | 39 % |
| **NGO Effectiveness (1-7)** | 4.22 (1.30) | 4.15  (1.29) | 4.22  (1.30) |
| **NGO Objectives (1-7)** | 4.17 (1.22) | 4.01  (1.27) | 4.19  (1.21) |
| **Meat Effect on Health (1-7)** | 3.81 (1.31) | 3.77  (1.32) | 3.86  (1.25) |
| **Meat Consumption (1-13)** | 7.01 (2.17) | 6.86  (2.20) | 7.18  (2.15) |
| **Tax Effect on Export (1-7)** | 4.69 (1.16) | 4.59  (1.23) | 4.72  (1.14) |
| **Tax Effect on Consump. (1-7)** | 4.37  (1.28) | 4.31  (1.36) | 4.34  (1.26) |
| **Government Effectiveness (1-7)** | 3.31  (1.30) | 3.07  (1.33) | 3.27  (1.29) |

**Appendix C**

Table 3.1: Results across different model specifications


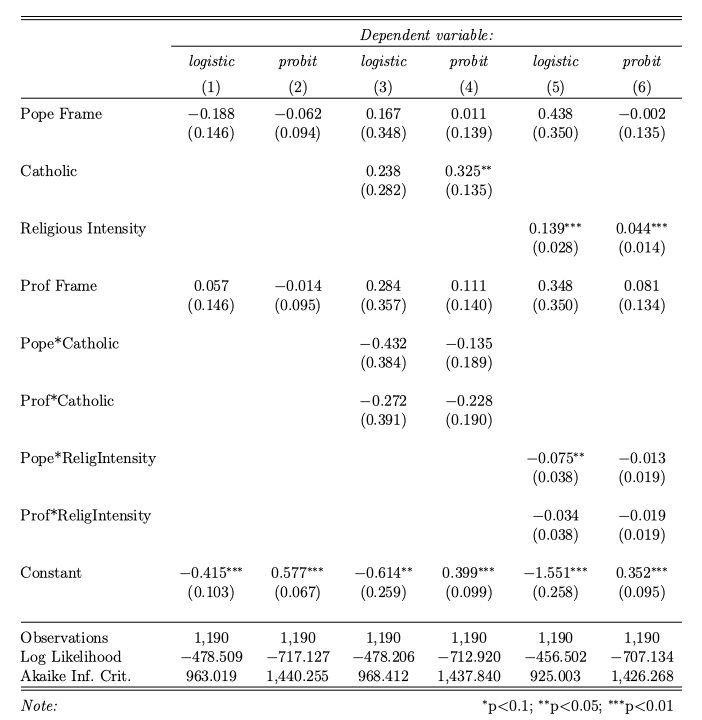


**Appendix**

**Table 3.1 Main Results with Variables for Alternative Mechanism**

**
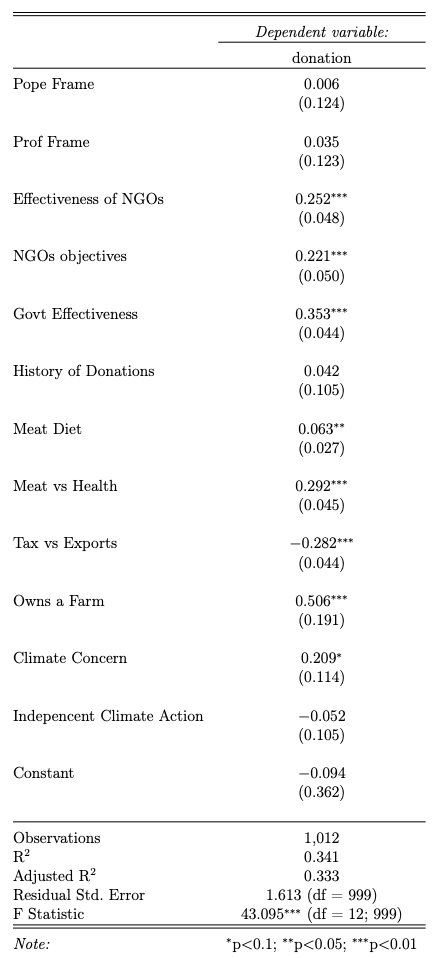
**
